# Tabular deep learning: a comparative study applied to multi-task genome-wide prediction

**DOI:** 10.1186/s12859-024-05940-1

**Published:** 2024-10-04

**Authors:** Yuhua Fan, Patrik Waldmann

**Affiliations:** Research Unit of Mathematical Sciences, University of Oulu, P.O. Box 8000, 90014 Univesity of Oulu, Finland

**Keywords:** Tabular data, Multi-trait, Genome-wide prediction (GWP), Non-linear models

## Abstract

**Purpose:**

More accurate prediction of phenotype traits can increase the success of genomic selection in both plant and animal breeding studies and provide more reliable disease risk prediction in humans. Traditional approaches typically use regression models based on linear assumptions between the genetic markers and the traits of interest. Non-linear models have been considered as an alternative tool for modeling genomic interactions (i.e. non-additive effects) and other subtle non-linear patterns between markers and phenotype. Deep learning has become a state-of-the-art non-linear prediction method for sound, image and language data. However, genomic data is better represented in a tabular format. The existing literature on deep learning for tabular data proposes a wide range of novel architectures and reports successful results on various datasets. Tabular deep learning applications in genome-wide prediction (GWP) are still rare. In this work, we perform an overview of the main families of recent deep learning architectures for tabular data and apply them to multi-trait regression and multi-class classification for GWP on real gene datasets.

**Methods:**

The study involves an extensive overview of recent deep learning architectures for tabular data learning: NODE, TabNet, TabR, TabTransformer, FT-Transformer, AutoInt, GANDALF, SAINT and LassoNet. These architectures are applied to multi-trait GWP. Comprehensive benchmarks of various tabular deep learning methods are conducted to identify best practices and determine their effectiveness compared to traditional methods.

**Results:**

Extensive experimental results on several genomic datasets (three for multi-trait regression and two for multi-class classification) highlight LassoNet as a standout performer, surpassing both other tabular deep learning models and the highly efficient tree based LightGBM method in terms of both best prediction accuracy and computing efficiency.

**Conclusion:**

Through series of evaluations on real-world genomic datasets, the study identifies LassoNet as a standout performer, surpassing decision tree methods like LightGBM and other tabular deep learning architectures in terms of both predictive accuracy and computing efficiency. Moreover, the inherent variable selection property of LassoNet provides a systematic way to find important genetic markers that contribute to phenotype expression.

## Background

Genome-wide prediction has become widespread as a valuable tool to estimate genetic merit in animal and plant breeding [1, 2]. In humans, GWP has been widely used to predict disease risk of highly polygenic complex human traits [3]. Genomic data is usually represented in matrices with markers in columns and individual observations in rows. Linear mixed models for GWP either model the markers effects directly or via pedigree based covariance models and both approaches often obtain competitive results [4, 5], but comparative studies show that machine learning (ML) methods are better in modelling various interactions in the genome [6]. In recent years, deep neural networks have emerged as powerful tools across various domains, ranging from sound and image analysis to natural language processing [7]. In these problems, the data points are represented as vectors of structured homogeneous features. However, in many other real-world applications, tabular data remains the most prevalent data type, consisting of samples in rows and different features in columns that don’t follow any simple structure [8, 9]. Over the past decade, numerous supervised, self-supervised, and semi-supervised learning methods have been proposed to address the specific challenges associated with modeling tabular data.

Decision tree models represent a widely adopted machine learning technique and serve as the foundation for more sophisticated ensemble methods such as random forests [10] and gradient boost decision trees (GBDT) [11–13]. Classical machine learning approaches, including GBDT, have dominated tabular data applications due to their superior performance. Various GBDT methods, such as XGBoost [11], LightGBM [12], and CatBoost [13], construct a robust predictor by ensembling weaker predictors through gradient descent in a functional space. They have been applied with success across diverse domains [14–16]. Despite their ability to model non-linear dependencies, decision trees struggle with out-of-distribution samples [17]. Furthermore, these methods lack flexibility and ability to utilize pre-trained models, thereby limiting their utility. However, they are computationally very efficient and can be utilized on big data sets.

In the context of genomics, a lot of research effort has been invested into prediction of molecular properties of organisms [18]. Single-layer neural networks have been utilized in animal and plant breeding [19, 20]. These shallow neural networks have been found to be prone to overfitting, although they are sometimes competitive with penalized linear models. Recently, several applications of GWP using deep learning have been studied and compared with other methods [21, 22]. The most commonly employed deep learning architectures in genomic prediction are the multi-layer perceptron (MLP) and one-dimensional convolutional neural networks (CNNs), which have been used for GWP in for example humans [23], pigs [24], wheat [25, 26] and soybean [27]. Current research has demonstrated that the CNN-RNN method exhibit superior performance compared to models based on random forest, deep fully connected neural networks, and conventional Lasso regression [28]. A deep neural network was designed to predict maize yield across 2,247 locations from 2008 to 2016 using both genotype and environmental data in the yield testing stage [29]. Furthermore, a number of studies have used multi-omics data to predict complex traits in various organisms, including animals, humans and plants [30–32].

The tabular representation of genomic data offers a structured framework for organizing, analyzing, and interpreting complex biological information [33]. Except for the usually underperforming MLP, few tabular deep learning models have been applied to GWP tasks. One exception is the GPtransformer which is a Transformer-based deep learning algorithm that was applied to predicting Fusarium related traits in Barley [34]. Neural networks offer an end-to-end pipeline of hierarchical feature extractors and a final estimator for tabular data, with components trained together to minimize loss functions using gradient-based strategies. Unlike decision trees, neural networks can to some extent maintain performance on out-of-distribution data [17]. Recent research has highlighted deep learning as a promising alternative approach to decision trees [9]. Based on Lasso regularization, three prominent models, the CNNGWP [24], the LassoNet [35] and the Neural Lasso [36], underscore the flexibility and efficiency of regularized deep learning methods. Developed by researchers at Google Cloud AI, TabNet [37] leverages the advantages of decision trees and attention mechanisms to effectively learn from tabular data, making it well-suited for tasks such as classification and regression. Additionally, NODE [38] combines neural oblivious decision trees with dense connections, applying the ensemble method of oblivious decision trees to neural networks using differentiable trees based on the entmax function. TabR [39] introduces a retrieval-augmented tabular deep learning architecture, achieving strong performance through attention-based retrieval components. AutoInt [40] reduces data sparsity by transforming high-dimensional data into a low-dimensional space using an embedding layer with a gating mechanism for feature representation learning and feature selection. Moreover, GANDALF [41] proposes a gated adaptive network for deep automated feature learning, incorporating a gating mechanism for feature representation learning. Tab-Transformer [42] utilizes self-attention based transformers to transform categorical feature embeddings into robust contextual embeddings for improved prediction accuracy. FT-Transformer [43], adapted from the transformer architecture, embeds all features before applying a stack of transformer layers to operate on the feature level, specifically designed for tabular data. By leveraging several mechanisms to overcome the difficulties of training on tabular data, SAINT [44] was proposed to boost performance for semi-supervised problems with a self-attention and intersample attention transformer.

Compared to typical linear statistical models, multi-task deep learning for genomic prediction tasks presents a more flexible and powerful framework by leveraging feature representation learning and facilitating learning across tasks. Although multi-task deep learning models perform well for classification or regression tasks on homogeneous input data (e.g., image, audio, and text data), its application to tabular genomic data still pose a challenge in the high-dimensional setting where the number of feature variables is considerably higher than the number of observations. In this paper, we present a comparative GWP study of tabular deep learning together with LightGBM as a GBDT representative using five real datasets for both regression and classification.

## Results

### Model comparison

**Multi-trait regression:** The results from the regression tasks are summarized in the Table  1. The comparative analysis of different models for multi-trait regression tasks across three datasets-Mice, Pig, and Wheat-reveals notable variations in performance, primarily measured by Mean Squared Error (MSE) and correlation coefficient (*r*). LassoNet consistently outperforms other models, achieving the lowest MSE and highest correlation across all datasets (e.g., Mice data: MSE = 0.135 ± 0.003, *r* = 0.735; Pig data: MSE = 0.151 ± 0.002, *r* = 0.722). This indicates LassoNet’s superior ability to capture the important information in the underlying data structure. In contrast, models like NODE and TabR show relatively poor performance, with higher MSE and lower correlation values, indicating less accurate predictions (e.g., NODE on Pig data: MSE = 0.242 ± 0.007, *r* = 0.531). GANDALF demonstrates competitive results, especially in the Wheat dataset, where it records an MSE of 0.191 ± 0.004 and a correlation of 0.651, close to LassoNet’s performance. The TabNet models, both supervised and unsupervised, perform moderately well across the datasets, with supervised TabNet slightly outperforming the unsupervised version. For instance, on the Pig data, supervised TabNet achieves an MSE of 0.225 ± 0.006 and *r* = 0.607, whereas the unsupervised version shows a slightly higher MSE of 0.243 ± 0.004 and *r* = 0.621. FT-Transformer, Tab-Transformer and SAINT offer a middle ground in terms of performance, with MSE values consistently lower than NODE but higher than LassoNet, reflecting a decent trade-off between complexity and predictive power. The results clearly demonstrate that LassoNet is the most effective model for multi-trait regression tasks over these datasets.

**Multi-class classification:** The experimental results for multi-class classification for GWD are reported in Table 2. In the multi-trait classification tasks, the performance of various models was evaluated based on their accuracy, area under the curve (AUC), and Brier score across two datasets: the 14-cancer microarray data and the Loblolly pine data (subset). Among the models, LassoNet once again emerged as the top performer, achieving the highest accuracy (90.1 ± 0.17) and AUC (91.5 ± 0.152) on the microarray data, and further outperforming other models on the Loblolly pine data with an accuracy of 93.5 ± 0.19 and an AUC of 96.7 ± 0.190, coupled with the lowest Brier scores (0.174 and 0.183, respectively). It is clear that LassoNet has strong capability in both predictive power and reliability. In contrast, TabNet (unsupervised) displayed the weakest performance, with the lowest accuracy (81.2 ± 0.37) and AUC (84.5 ± 0.205) on the microarray data as well as worse metrics on the Loblolly pine data (accuracy of 90.3 ± 0.25 and AUC of 93.6 ± 0.233). This highlights the challenges faced by unsupervised approaches in these prediction tasks. GANDALF and FT-Transformer also showed competitive results, with GANDALF reaching an accuracy of 87.4 ± 0.33 and an AUC of 89.7 ± 0.163 on the microarray data. On the Loblolly pine data the accuracy was 91.5 ± 0.26 and AUC 95.1 ± 0.194, indicating relatively robust performance. FT-Transformer also positioned itself as a strong contender.

**Fig. 1:**
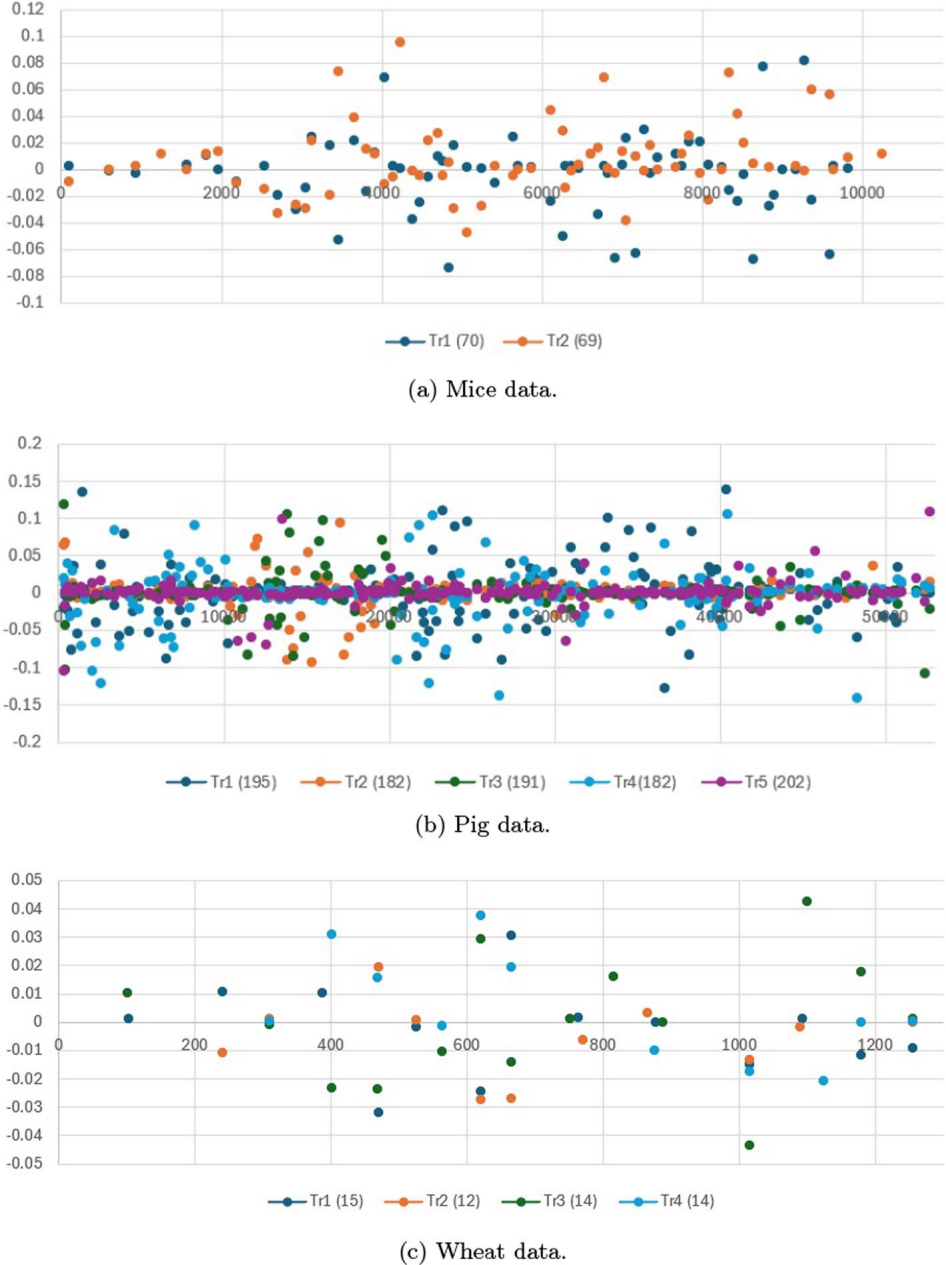
Selected coefficients using LassoNet for multi-trait regression over three datasets, with the number of features selected on a specific trait noted in parentheses (zero values in the coefficient matrix have been removed)

**Fig. 2:**
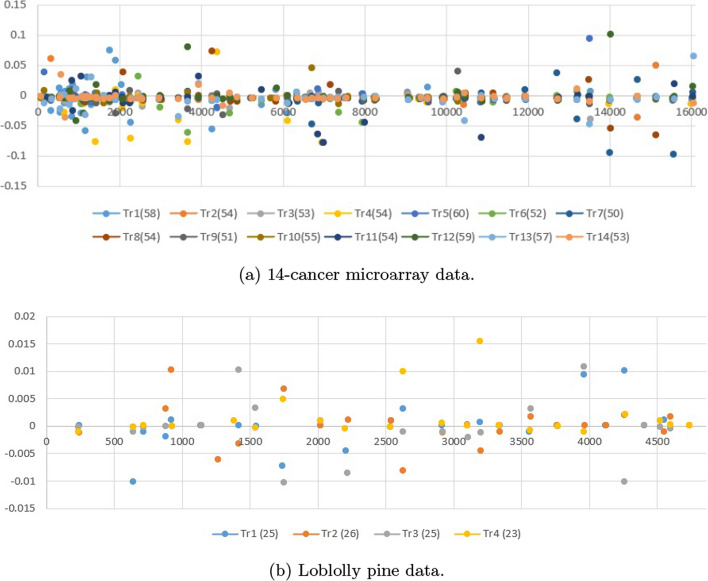
Selected coefficients using LassoNet over two datasets for multi-class classification, with the number of features selected on a specific task noted in parentheses (zero values in the coefficient matrix have been removed)

### Interpretability analysis

In the context of multi-trait genomic prediction, interpretability of models is becoming an increasingly important issue for understanding the influence of specific genomic features on model predictions [45, 46]. Feature selection of input variables provides a straightforward interprebablity of the evaluated models in this study. Compared with other methods, LassoNet offers a high interpretability due to its use of $L_1$ regularization (Lasso penalty), which by its inherent sparsity operation identify which input features are most relevant for making predictions (139 on the mice data, 952 on the pig data, 55 on the wheat data, 764 on the 14-cancer microarray data, and 99 on the Loblolly pine data). By selecting a subset of features, LassoNet can focus on the most influential variables while ignoring irrelevant or redundant ones. LightGBM can also provide interpretable results in terms of feature importance and decision paths within the trees. However, LightGBM does not rely on $L_1$ regularization for interpretation, but instead provides two different types of feature importance: split and gain importance. On the other side, the tabular deep learning methods lack the inherent interpretabily due to their more complex neural network structures. This complexity enables them to capture the patterns present in genomic data but also make interpretation and understanding of model decision more challenging. We only visualize the selected coefficients for the five datasets using LassoNet due to the superiority of this model. Figures 1 and 2 shows the selected coefficients for multi-trait regression and multi-class classification over five datasets.

**Table 1:** Comparison of model performance across different datasets for multi-trait regression tasks

	Mice data		Pig data		Wheat data	
Model	MSE (mean ± stddev)	r	MSE (mean ± stddev)	r	MSE (mean ± stddev)	r
LightGMB	0.208 ± 0.005	0.572	0.203 ± 0.006	0.652	0.160 ± 0.004	0.657
NODE	0.231 ± 0.008	0.501	0.242 ± 0.007	0.531	0.223 ± 0.006	0.514
LassoNet	**0.135 ± 0.003**	**0.735**	**0.151 ± 0.002**	**0.722**	**0.149 ± 0.003**	**0.691**
AutoInt	0.253 ± 0.007	0.571	0.267 ± 0.006	0.633	0.228 ± 0.005	0.641
TabNet (unsupervised)	0.301 ± 0.006	0.566	0.243 ± 0.004	0.621	0.218 ± 0.004	0.635
TabNet (supervised)	0.252 ± 0.008	0.554	0.225 ± 0.006	0.607	0.216 ± 0.005	0.626
FT-Transformer	0.221 ± 0.005	0.531	0.219 ± 0.004	0.585	0.206 ± 0.004	0.602
Tab-Transformer	0.223 ± 0.006	0.526	0.221 ± 0.005	0.563	0.209 ± 0.005	0.558
TabR	0.241 ± 0.009	0.503	0.230 ± 0.007	0.534	0.211 ± 0.006	0.541
GANDALF	0.211 ± 0.006	0.571	0.214 ± 0.005	0.637	0.191 ± 0.004	0.651
SAINT	0.224 ± 0.007	0.537	0.227 ± 0.006	0.581	0.210 ± 0.005	0.576

**Bold** indicates the best results

**Table 2:** Comparison of different models for multi-class classification tasks on two datasets

	14-cancer microarray data			Loblolly pine data (subset)		
Model	Accuracy (mean ± stddev)	AUC (mean ± stddev)	Brier score	Accuracy (mean ± stddev)	AUC (mean ± stddev)	Brier score
LightGMB	$85.7 \pm 0.32$	88.9 ± 0.171	0.202	$91.5 \pm 0.27$	93.7 ± 0.201	0.206
NODE	$85.1 \pm 0.35$	88.7 ± 0.237	0.243	$91.1 \pm 0.31$	93.4 ± 0.223	0.301
LassoNet	**90.1 ± 0.17**	**91.5 ± 0.152**	**0.174**	**93.5 ± 0.19**	**96.7 ± 0.190**	**0.183**
AutoInt	$85.2 \pm 0.33$	88.6 ± 0.263	0.381	$91.5 \pm 0.30$	94.3 ± 0.207	0.372
TabNet (unsupervised)	$81.2 \pm 0.37$	84.5 ± 0.205	0.406	$90.3 \pm 0.25$	93.6 ± 0.233	0.383
TabNet (supervised)	$82.3 \pm 0.37$	86.1 ± 0.207	0.355	$90.1 \pm 0.33$	93.6 ± 0.246	0.361
FT-Transformer	$84.7 \pm 0.34$	88.8 ± 0.181	0.235	$90.8 \pm 0.34$	94.1 ± 0.252	0.245
Tab-Transformer	$83.4 \pm 0.34$	88.5 ± 0.177	0.238	$90.6 \pm 0.32$	94.0 ± 0.243	0.253
TabR	$85.1 \pm 0.35$	88.6 ± 0.183	0.244	$91.4 \pm 0.27$	93.5 ± 0.221	0.355
GANDALF	$87.4 \pm 0.33$	89.7 ± 0.163	0.227	$91.5 \pm 0.26$	95.1 ± 0.194	0.241
SAINT	$84.6 \pm 0.32$	88.8 ± 0.175	0.241	$90.7 \pm 0.27$	93.5 ± 0.185	0.263

**Bold** indicates the best results

**Table 3:** Time report in seconds (s) (mean ± stddev) of one BO iteration using 5 GPUs for different models on the five data sets

	Data sets				
Model	Mice data (s)	Pig data (s)	Wheat data (s)	14-cancer microarray data (s)	Loblolly pine data (subset) (s)
LightGBM	**4.9 ± 0.4**	**33.2 ± 3.5**	**25 ± 2.6**	**8.4 ± 0.52**	**4.1 ± 0.27**
NODE	46.3 ± 6.3	207 ± 11.7	240 ± 14.3	33.1 ± 3.7	9.1 ± 2.1
LassoNet	5.6 ± 1.1	36.1 ± 3.3	32 ± 3.6	8.8 ± 2.7	**4.1 ± 1.1**
AutoInt	9.3 ± 1.7	56.8 ± 4.4	53 ± 3.6	9.1 ± 2.2	4.4 ± 1.2
TabNet (unsupervised)	19.1 ± 5.1	75.5 ± 4.3	123 ± 9.1	10.9 ± 2.3	5.7 ± 1.7
TabNet (semi-supervised)	16.9 ± 5.4	67.3 ± 6.1	113 ± 7.7	10.1 ± 4.1	5.2 ± 1.3
FT-Transformer	14.1 ± 3.9	62.3 ± 5.6	147 ± 10.4	13.7 ± 4.0	6.8 ± 2.2
Tab-Transformer	16.3 ± 3.6	85.3 ± 4.8	93 ± 6.6	11.2 ± 5.3	5.9 ± 0.9
Tab-R	55.1 ± 5.5	175 ± 10.7	271 ± 11.6	35.4 ± 7.1	11.2 ± 2.3
GANDALF	16.5 ± 3.1	52.3 ± 6.2	98 ± 5.7	10.2 ± 2.3	5.0 ± 1.0
SAINT	15.3 ± 2.1	67.1 ± 4.2	97.3 ± 4.9	14.1 ± 3.7	7.5 ± 2.1

**Bold** indicates the best results

### Computation time

To improve the computing efficiency, we use parallel computing with 5 GPUs for the different models. The time required for one BO iteration of the models are summarized in Table 3. We can clearly see that LightGBM consistently outperforms other models, achieving the fastest execution times across all datasets. For instance, LightGBM completes one Bayesian Optimization (BO) iteration on the Mice dataset in only 4.9 ± 0.4 seconds (s), significantly faster than any other model. Similarly, it maintains a clear advantage on larger datasets such as Pig, Wheat, and 14-cancer microarray data, with times of 33.2 ± 3.5, 25 ± 2.6, and 8.4 ± 0.52 s, respectively. In contrast, NODE and Tab-R exhibit the slowest performance, particularly on the Pig and Wheat datasets where NODE takes 207 ± 11.7 and 240 ± 14.3 s, respectively, and Tab-R requires 175 ± 10.7 and 271 ± 11.6 s. This indicates that these models are not optimized for computational speed, especially in a multi-GPU setting. LassoNet and AutoInt show competitive performance with LassoNet nearly matching LightGBM on the Loblolly pine dataset, finishing in 4.1 ± 1.1 s. However, they generally lag behind LightGBM on the other datasets. Models like TabNet (both unsupervised and semi-supervised), FT-Transformer, Tab-Transformer, and SAINT offer moderate performance, with execution times ranging between those of the fastest and slowest models. These models, while not as fast as LightGBM, are notably quicker than NODE and Tab-R, particularly on smaller datasets like the Mice and 14-cancer microarray data.

## Discussion

In one earlier study [47], we evaluated several linear sparse proximal multi-task learning (SPMTL) methods on the same genomic datasets as in the current study. The regularization methods there include Lasso, group Lasso, sparse group Lasso, nuclear norm, and the $L_{2,\frac{1}{2}}$ norm. Among the evaluated methods, the $L_{2,\frac{1}{2}}$ norm achieved the best out-of-sample prediction performance across all datasets with the smallest test MSE and the highest *r* on the mice data $(\textrm{MSE}=0.151, r=0.702)$, pig data $(\textrm{MSE}=0.160, r=0.71)$, and wheat data $(\textrm{MSE}=0.180, r=0.620)$.

Compared with $L_{2,\frac{1}{2}}$ norm from SPMTL methods, the current study presents an obvious reduction in test MSE with LassoNet across three datasets: a decrease of 10.6% for the mice datasets, 5.7% for the pig data, and 17.2% for the wheat dataset, respectively. Moreover, employing Markov Chain Monte Carlo (MCMC)-based multi-task Bayesian ridge regression (BRR) and Bayesian spike-and-slab analysis further confirms the efficacy of LassoNet. Specially, the test MSE exhibits significant improvements with a reduction of 48.8% compared to BRR on the mice dataset, 50.3% compared to spike-and-slab on the pig dataset, and 43.5% compared to BRR on the wheat data. These results demonstrate that LassoNet is very competitive in comparison to both linear SPMTL methods, Bayesian GWP methods and other tabular neural network methods in performing joint predictions across correlated traits.

In [48], linear classifiers with eight different regularization methods were assessed on the microarray data that contains 14 cancer classes. These techniques included nearest shrunken centroids, $L_2$-penalized discriminant analysis, support vector classifier, Lasso regression (one vs all), *k*-nearest neighbors, $L_1$/$L_2$ penalized multinomial, and elastic-net penalized multinomial regression. Among these methods, the elastic-net penalized multinomial method achieved the highest accuracy of 78.1%. In contrast, LassoNet attained an accuracy of 90.1% on the same 14-cancer microarray data, clearly outperforming the evaluated linear classifiers employing different regularization approaches in facilitating joint predictions across multiple traits.

LassoNet can capture complex interaction effects between features via its neural network module. When input features are correlated or interact with each other in predicting the target phenotype, LassoNet assigns non-zero coefficients to these features to account for their joint effect. However, because of the model architecture and the regularization that encourage sparsity, LassoNet’s ability to capture interactions is smaller compared to the interaction-based models like AutoInt, TabR and TabNet. Additionally, the evaluations indicate that some tabular deep learning models can achieve comparable or competitive results in classification tasks across our datasets (GANDALF). For the models based on transformers (AutoInt, TabTransformer, TabNet and SAINT), we can notice that a self-attention mechanism within the encoder can capture more complex interactions, but it doesn’t improve results compared to LassoNet since they lack efficient regularization procedures.

## Conclusion

In this paper, we provide an overview and comparison of recent deep learning architectures tailored for tabular data and their application to multi-trait genome-wide prediction (MTGWP). Through extensive evaluations on real-world genomic datasets, the study identifies LassoNet as a standout performer, surpassing decision tree methods like LightGBM and other tabular deep learning architectures in terms of both predictive accuracy and computing efficiency. Moreover, the inherent variable selection property of LassoNet provides a systematic way to find important genetic markers that contribute to phenotype expression.

## Methods

### Definitions

The key part of tabular deep learning is a deep neural network and usually in the form of a feed-forward network. A deep neural network defines a mapping $\hat{f}$ [7] following

1 $$\begin{aligned} Y = f(X) \approx \hat{f}(X; W), \end{aligned}$$

that learns the model parameters *W* (i.e. the weights of a neural network) that results in the best approximation of true underlying and unknown function *f*. In this case, $X \in \mathcal {R}^{p}$ is the input feature data with corresponding output target $Y \in \mathcal {Z}^{k}$ for *k* classes or $Y \in \mathcal {R}^{k}$ for *k* regression tasks represented as a set of tuples $\{x_i, y_i\}_{i\in n}$. Throughout this study, we focus on genomic data *X* of dimension $n \times p$ with associated multiple traits *Y* of dimension $n \times k$ with the restriction that the all traits are of the same modality (i.e. either continuous or binary). However, the input data can be of different modalities (for example a mixture between DNA marker and RNA expression data) which fundamentally contrasts with the homogeneous nature of image, audio or text data. The network used in this work is feed-forward, which means that the input information flows in one direction to the output without any feedback connections [7]. Multi-task tabular deep learning involves training a model to simultaneously minimize a multivariate loss function $\mathcal {L}(\hat{f}(X; W), Y)$. The goal is to capitalize on possible shared representations across related tasks in order to enhance the model’s performance.

### Tabular neural networks

#### NODE

Inspired by CatBoost [13], the neural oblivious decision ensemble (NODE) [38] is designed to leverage the interpretability of decision trees while benefiting from the expressive prediction power of neural networks.

Each oblivious decision module in NODE consists of several layers of decision nodes. At each layer *l*, each node makes a decision based on a splitting feature *g* and a learnable threshold *b*. For the *l*-th layer and node *j*, the decision function can be expressed as

2 $$\begin{aligned} d_{l,j}(X)=\mathcal {H}(g_{l,j}-b_{l,j}), \end{aligned}$$

where $\mathcal {H}(\cdot )$ denotes the Heaviside step function and $d_{l,j}(X)$ is the output of the decision node. Then all *r* decision nodes in the *l*-th layer will produce a decision vector $v_{l} = [d_{l,1}(X), d_{l,2}(X), \cdots , d_{l,r}(X)]$. NODE uses an ensemble of such decision trees through these output nodes.

To make the oblivious decision trees (ODTs) differentiable, the splitting feature choice $g_{l,j}$ and the comparison operator $\mathcal {H}(g_{l,j}-b_{l,j})$ are replaced by their differentiable counterparts. The choice function is replaced by a weighted sum of features, with weights computed based on the $\alpha -\text {entmax}$ transformation [49] over the learnable feature selection matrix. The Heaviside function $\mathcal {H}(\cdot )$ is relaxed to a two-class $\text {entmax}$, which is denoted as $\sigma _{\alpha }(v) = \text {entmax}_{\alpha }([v, 0])$. Its scaled version $c_{l,j}(X) = \sigma _{\alpha }(\frac{g_{l,j}(v) - b_{l,j}}{\tau _{l,j}})$ is used due to potential variations in feature scales by using learnable parameters. The computed $c_{l,j}(v)$ is combined into a “choice” tensor $C \in \mathcal {R}^{2^{d}}$. The final prediction is then computed as a weighted linear combination of response tensor entries *R* with weights from the entries of choice tensor *C*. Assume that the tree outputs are one-dimensional $\hat{h}(v)$ and each NODE layer contains several trees whose outputs are concatenated by *m* individual trees $[\hat{h}_{1}(v), \cdots , \hat{h}_{m}(v)]$. Then the NODE layer can be trained alone or within a complex structure, just like fully-connected layers that serve as input for the subsequent layers. Similar to DenseNet [50], this architecture is a sequence of *l* NODE layers, where each layer uses a concatenation of previous layers as its input. This aggregated output is then passed through a final fully connected layer to produce the final prediction $\hat{Y}$, with the structure of this final layer depending on the task (regression or classification).

#### TabR

TabR [39] is a feed-forward network incorporating a customized *t*-Nearest-Neighbors-like component in the middle layer to produce a better prediction. Its main idea is to utilize the self-attention mechanism of transformers to capture complex interactions between features in tabular data.

With the feature matrix *X*, a feed-forward retrieval-free network $f(X)=P(E(X))$ is first partitioned into two parts: an encode $E:X\rightarrow \mathcal {R}^{p^{'}}$ part and a predictor $P: \mathcal {R}^{p^{'}}\rightarrow P{\hat{Y}}$ part. To make the model incrementally retrieval-based, a retrieval module *R* in a residual branch is added after *E*, where $\tilde{X} \in \mathcal {R}^{p^{'}}$ is the intermediate representation of the target object, $\{\tilde{x}_{i}\}_{i\in I_{cand}} \subset \mathcal {R}^{p^{'}}$ are the intermediate representations of the candidates and $\{y_{i}\}_{i\in I_{cand}}\subset Y$ are the labels of the candidates.

The retrieval module *R* is defined in the spirit of *k*-nearest neighbors. For the target object’s representation, the retrieval module takes the $x_{1,...,t}$ nearest neighbors among the candidates $\tilde{x}_{i}$ according to the similarity module $\mathcal {S}$ and aggregates their values produced by the value module $\mathcal {V}$ with the definitions

3 $$\begin{aligned} \mathcal {S}(\tilde{X},\tilde{x}_{i}) = W_{Q}(\tilde{X})^{T}W_{K}(\tilde{x}_{i}) \quad \quad \mathcal {V}(\tilde{X},\tilde{x}_{i},y_{i}) = W_{V}(\tilde{x}_{i}), \end{aligned}$$

where $W_{Q}$, $W_{K}$, $W_{V}$ are the weights for the corresponding transformation. They play a critical role in transforming inputs to better capturing the similarities between entries, contributing to the model’s ability to learn complex patterns and relationships within the data. By adding context labels, the performance of the similarity $\mathcal {S}$ and the value module $\mathcal {V}$ can be improved. Finally, the formal complete description of TabR which implements the *R* module is

4 $$\begin{aligned} \mathcal {S}(\tilde{X},\tilde{x}_{i}) = -\left\| t-t_{i} \right\| ^{2} \quad \quad \mathcal {V}(\tilde{X},\tilde{x}_{i},y_{i}) = W_{y_{i}} + O(t-t_{i}), \end{aligned}$$

where $t=W_{K}(\tilde{X})$, $t_{i}= W_{K}(\tilde{x}_{i})$and the operation of *O* is defined as $O(\cdot )=\text {LinearWithoutBias}(\text {Dropout}(\text {Relu}(\text {Linear}(\cdot ))))$. The retrieval module *R* enriches the target object’s representation by retrieving and processing relevant objects from the candidates. Finally, the predictor *P* makes a prediction.

#### TabNet

TabNet [37] combines the strengths of both tree-based methods and deep neural networks using a sequential attention mechanism. It emerges as a deep learning model embodying the feature selection principles of decision trees, with its encoder comprising a feature transformer, an attentive transformer, and feature masking.

The features in *X* will be the input to a batch normalization layer which yields $X^{'} \in \mathcal {R}^{w \times z}$, where *w* is the batch size and *z* is the dimension. Assume the number of hidden layers is *l*, the output of the feature transformer then becomes $w \times l$ which is split into two parts to construct a gated linear unit: a standard decision step $\rho [i]\in \mathcal {R}^{w \times l_{a}}$ and a shared across decision step $a[i] \in \mathcal {R}^{w \times l_{\rho }}$. The former is used for the final output of TabNet, and the latter is used as an input of the attentive transformer. Each block is composed of a fully-connected (FC) layer, batch normalization (BN) and a gated linear unit (GLU). For the attentive transformer, the main function is to get a learnable mask layer $M[j] \in \mathcal {R}^{w \times z}$ according to

5 $$\begin{aligned} M[j] = \text {sparsemax}(P[j-1] \cdot \gamma _{i}(a[j-1])), \end{aligned}$$

where $\gamma _{i}(a[i-1]))$ is from the FC to BN, and *sparsemax* is a mapping from the vector to a simplex that obtains sparsity. The scaling prior - *P*[*j-1*] has a close connection to the mask *M*[*j*] via the features used in previous steps and one can notice that the initial value of *P*[0] equals 1. To ensure the sparsity of *M*[*j*], a regularized constraint is given to the parameters to make the distribution of *M*[*j*] more reasonable.

All the output from the earlier steps are summed to give the final output through the FC layer. For the multi-task learning, each task-specific branch ends with an output layer that produces a scalar output for the regression task. The shared layers can facilitate the extraction of relevant features for multi-task learning and the task-specific branches capture patterns specific to each multi-task regression target. The TabNet decoder is composed of a feature transformer block at each step. After reconstructing the features from the encoded representation, the aggregated features will be passed through a fully connected layer to do the predictions $\hat{Y}$.

#### TabTransformer

Considering the characteristics of context embeddings, TabTransformer [42] is built upon self-attention based transformers. This model comprises a column embedding layer, a stack of *l* Transformer layers, and a multi-layer perceptron. Each Transformer layer consists of a multi-head self-attention layer followed by a position-wise feed-forward layer. For the tuples $\{x_{i}, y_{i}\}_{i\in n}$, each of the $x_{i}$ is embedded into a parametric embedding of dimension *s* using column embedding. Let $e_{\phi _{i}}(x_{i}) \in \mathcal {R}^{s}$ be the embedding of the $x_i$ feature, and $E_{\phi }(x_{cat}) = \{e_{\phi _{1}}(x_{1}), \cdots , e_{\phi _{s^{'}}}(x_{s^{'}})\}$ is the set of embeddings for all the categorical features. Then $E_{\phi }(x_{cat})$ serves as input to the sequential Transformer layers $f_{\theta }$, which operate on parametric embeddings and return the corresponding contextual embeddings $h_{s^{'}}$ where $h\in \mathcal {R}^{s}$. These contextual embeddings are concatenated along with the features to first form a vector which serves as the input to an MLP that is used to predict the target $\hat{Y}$.

A self-attention layer in TabTransformer comprises three parametric matrices - Key (*K*), Query (*Q*) and Value (*V*). Each input embedding is projected on to these matrices to generate the corresponding vectors and attends to all other embeddings through an attention head, which is computed as $\text {Attention}(K, Q, V) = A \cdot V$ , where $A = \text {softmax}((QK^{T})/\sqrt{k {'}})$($k^{'}$ is the dimension of Key). The output of the attention head is projected back to the embedding through a FC, which in turn is passed through two position-wise feed-forward layers. The contextual embeddings are concatenated to form the feature $x_{cont}$. If we let $\delta$ be the cross-entropy for classification and the mean square error for regression tasks, the prediction $\hat{Y}$ can be obtained by minimizing the loss function $\mathcal {L}(\hat{f}(X;W), Y)=\delta (MLP(\text {Transformer}(E_{\phi }(x_{cat})),x_{cont}),Y)$.

#### FT-Transformer

FT-Transformer performs feature transformations that enhance the model’s ability to capture complex patterns [43]. It handles individual features independently before combining them to make predictions. There are two important parts of FT-Transformer: the feature tokenizer and the transformer.

The feature Tokenizer component first transforms the input feature *X* to embeddings $G \in \mathcal {R}^{m^{'} \times n^{'}}$. The embedding for the feature $x_i$ is computed as

6 $$\begin{aligned} G_{i} = f_{i}(x_i) + b_{i}, \end{aligned}$$

where $b_{i}$ is the *i*-th feature bias, $f_{i}$ is implemented as the element-wise multiplication with the weight matrix $W_{i}$. There is also a function $f_{i}^{(cat)}$ implemented as a lookup table $W_{i}^{(cat)}$ for categorical features with one-hot vectors of the corresponding categorical features $e_{i}^{T}$. Then, the vectors are stacked as $G = stack[G_{1}, \cdots , G_{i}, G_{1}^{(cat)}, \cdots , G_{i}^{(cat)}]$ and the embedding of the [CLS] token (or “output token”) is appended to the *G* and *l* Transformer layers $F_{1}, F_{2}, \cdots , F_{l}$ as

7 $$\begin{aligned} G_{0} = \text {stack}[[CLS], G] \quad \quad G_{i}=F_{i}(G_{i-1}). \end{aligned}$$

After using the PreNorm setting, the final representation of the [CLS] token is used for prediction. For the multi-task learning situation, the initial layer of FT-Transformer consists of a shared transform encoder that will process the input feature that are propagated as the task-specific heads for each regression task. These heads are small MLPs that take the output of the shared encoder and generate task-specific predictions. Denoting the final representation of the [*CLS*] token as $G_{l}^{[CLS]}$, then the prediction is $\hat{Y}=f(G_{l}^{[CLS]};W)$.

#### AutoInt

Given the limited ability of shallow networks to model interactions, AutoInt [40] is designed based on transformer mechanisms that enhance the modelling capabilities for feature interactions. The main idea of AutoInt is mapping of the original features to sparse low dimensions and modeling of the interactions among the high-order features.

With an embedding vector $\upsilon _{i}$ for field *i*, the original feature $x_i$ is embedded into low dense vectors through the embedding layer as $\sigma _{i} = \upsilon _{i} x_{i}$. The output of the embedding layer is a concatenation of multiple embedding vectors, which are the input of an interaction layer. For the following interaction layer, a multi-head mechanism is utilized to map the feature into multiple subspaces and generate the different feature interaction pattern in these spaces. Further on, more high-order interactions will be produced through stacking of interaction layers. For the feature $\sigma _{i}$ in attention space *I*, there are three vectors: $W_{Q}$ for query, $W_{K}$ for key, and $W_{V}$ for value, respectively. The similarity between the feature $\sigma _{i}$ and feature $\sigma _{j}$ is first obtained as $\phi ^{I} = <W_{Q}^{I}\sigma _{i}, W_{K}^{I}\sigma _{j}>$, and then the distribution of the attention is produced using softmax. With a weighted sum, the new feature of $\sigma _{i}$ can be acquired as $\hat{\sigma _{i}}^{I}$.

For multiple attention spaces, the new feature from each space can be concatenated to get the final representation of $\sigma _{i}$ as $\hat{\sigma }_{i}$. To preserve the learned combinatorial features, including raw individual features, a standard connection is added to the network

8 $$\begin{aligned} \sigma _{i}^{Res} = \text {ReLU}(\hat{\sigma }_{i} + W_{Res} \sigma _{i}), \end{aligned}$$

where $W_{Res}$ is the projection matrix and $\text {ReLU}(z) = \text {max}(0, z)$ is the standard non-linear activation function. Thus, the representation of each feature $\sigma _{i}$ is updated into a new representation $\sigma _{i}^{Res}$. By stacking multiple such layers, an arbitrary order of $\hat{\sigma }_{i}$ can be modeled. The output of the interacting layer is a set of feature vectors $\{\sigma _{i}^{Res}\}_{i=1}^{p}$. By concatenating all of the learned feature interactions, the aggregated representation will be passed through a final layer for the predictions of $\hat{Y}$.

#### LassoNet

LassoNet is based on the sparsity idea of the Lasso and achieves feature sparsity by allowing a feature to participate in a hidden unit only if its input connection is active [35]. The features *X* and a residual feed-forward neural network $\mathcal {F}$ with an arbitrary width and depth [51] can be described as

9 $$\begin{aligned} \mathcal {F} = \{\hat{f} \equiv \hat{f}_{\theta , W}: X \mapsto \theta ^{T}X + g_{W} X\}, \end{aligned}$$

where $g_{W}$ is a feed-forward network with wights *W* (fully connected). The object function of LassoNet for multi-task learning is

10 $$\begin{aligned} \arg \min \limits _{\theta , W} \mathcal {L}(\theta , W) + \lambda \left\| \theta \right\| _{1} \quad s. t. \quad \quad \left\| W_{i}^{(1)} \right\| _{\infty }\le \nu \left| \theta _{i} \right| , i=1,\cdots ,p, \end{aligned}$$

where $\mathcal {L}(\theta ,W)$ is the loss on the training data set, and $W_{i}^{1}$ denotes the weights for feature *i* in the first hidden layer. The constraint means that the total amount of non-linearity involving feature *i* according to the relative effect importance of $x_{i}$ as a main effect. The residual link and the first hidden layer jointly pass through a hierarchical soft-thresholding optimizer $\mathcal {S}(x)=sign(x)\cdot max\left\{ \left| x \right| - \lambda , 0 \right\}$. For the multi-task learning, the layers of the neural network remain common across all tasks to capture shared representations. The sparsity of the  input layer weights gives complete control of the feature sparsity of the network. When $\nu =0$, all the hidden units are inactive and only the skip connection remains which means that the formulation recovers exactly the Lasso. On the other hand, when $\nu \rightarrow \infty$, one recovers a standard unregularized feed-forward neural network. The linear and nonlinear components are optimized jointly to capture arbitrary nonlinearity.

#### GANDALF

Inspired by gated recurrent units (GRUs) [52] for representation learning, the gated adaptive network for deep automated learning of features (GANDALF) is designed for tabular data based on a gating mechanism and in-built feature selection called Gated Feature Learning Unit (GFLU) [41].

A learnable mask $M_n \in \mathcal {R}^{p}$ is used for the soft sparse selection of important features for each stage *n* of feature learning in GFLU. The mask is constructed by applying a sparse transformation on a learnable parameter vector $\Im _{n}\in \mathcal {R}^{p}$ combined with *t*-softmax activation for encouraging sparsity selection [53]. Here, let $X_{n}$ be the input features and $M_{n} = t\text {-softmax}(\Im _{n})$ the mask, the feature selection can be defined by

11 $$\begin{aligned} X_{n}&= M_n \odot X \end{aligned}$$

12 $$\begin{aligned} M_n&= t\text {-softmax}(\Im _{n}, t), \end{aligned}$$

where $\Im _{n}$ and *t* are learnable parameters and $\odot$ denotes an element-wise multiplication operation. The weight matrix *W* depends on the value of *t*. The gating mechanism has a reset gate $r_{n}$ and an update gate $z_n$. The update gate decides how much information to update in its internal feature representation, which can be defined as

13 $$\begin{aligned} z_n = \sigma (W_{n}^{z} \cdot [\varphi _{n-1}; x_{n}]), \end{aligned}$$

where $\varphi _{n-1}$ is the $(n-1)$-th stage of the GFLU and $W_{n}^{z}$ is a learnable parameter for the weight at stage *n*. Then the candidate feature representation $\hat{\varphi }_{n}$ is computed as

14 $$\begin{aligned} \hat{\varphi }_{n} = tanh(W_{n}^{O} \cdot [r_n \odot \varphi _{n-1}; X]), \end{aligned}$$

where $r_{n}$ decides how much information to forget from the previous feature representation, [] represents a concatenation operation, and $W_{n}^{O}$ represents a learnable parameter. The reset gate can be computed in a similar way as the update gate: $r-{n} = \sigma (W_{n}^{r}\cdot [\varphi _{n-1};X_{n}])$.

GANDALF can be viewed as a stack of GFLUs arranged in a sequence mannerthat at each stage *n* selects a subset of features and learns a representations of features and therefore multiple stages act in a hierarchical way to built up the optimal representation for the prediction task. Then this representation is fed to a multi-layer perceptron for the final prediction.

#### SAINT

SAINT (self-attention and intersample attention transformer) [44] is inspired by the transformer encoder, where the model takes in a sequence of feature embeddings and outputs contextual representations of the same dimension. Its main idea is to leverage several mechanisms to overcome the difficulties of training on tabular data. For the embedding layer, each feature in the input row is embedded into a *e*-dimensional space as

15 $$\begin{aligned} \textbf{E} = \text {Embedding}(X), \end{aligned}$$

where $\textbf{E}\in \mathbb {R}^{n\times p \times e}$, and *e* is the embedding dimension. In the stacking of *L* identical stages, each stage consists of one self-attention transformer block and one intersample attention transformer block. The contextual representations of the input of batch *b* can be given as $\{\mathbf {r_{i}}\}_{i=1}^{b} = \textbf{S}(\{\textbf{E}(x_{i})\}_{i=1}^{b})$. When $L=1$, $\mathbf {r_{i}}$ can be obtained as the following procedure

16 $$\begin{aligned} \begin{aligned} \textbf{z}_{i}^{(1)}&= \text {LN}(\text {MSA}(\textbf{E}(x_{i}))) + \textbf{E}(x_{i}) \\ \textbf{z}_{i}^{(2)}&= \text {LN}(\text {FF}_{1}(\textbf{z}_{i}^{(1)})) + \textbf{z}_{i}^{(1)}\\ \textbf{z}_{i}^{(3)}&= \text {LN}(\text {MISA}(\{\textbf{z}_{i}^{(2)}\}_{i=1}^{b})) + \textbf{z}_{i}^{(2)} \\ \textbf{r}_{i}&= \text {LN}(\text {FF}_{2}(\textbf{z}_{i}^{(3)})) + \textbf{z}_{i}^{(3)} \end{aligned}, \end{aligned}$$

where MSA is a multi-head self-attention layer with *h* heads, FF is a fully-connected feed-forward layer with a GELU non-linearity, LN is a normalization layer with skip connection and MISA is an intersample attention transformer block. For the intersample attention, it is computed across the different data points (i.e. rows of the tabular data matrix) in the batch. This can be helpful to improve the representation of a given data point by inspecting other points. For the self-supervised pretraining method, CutMix is used to augment samples in the input space and mixup is used in the embedding space for the augmented representation. At the final prediction stage, the corresponding embedding is passed through a single layer MLP with ReLU activation to get the output $\hat{Y}$.

### Implementation details

#### Tuning

For each dataset, we tuned the hyperparameters of each model using Bayesian optimization (BO) with 100 iterations. The hyperparameter search was conducted on the validation folds of the training set, ensuring that the test set remained untouched and independent. To optimize the hyperparameters, we used a 5-fold cross-validation (CV) approach on the training set. For each fold, the model was trained on 4 folds and validated on the remaining fold. This process was repeated 5 times, with each fold serving as the validation set once. The performance metrics from the 5 folds were averaged to obtain a single performance measure for the given set of hyperparameters. Various combinations of hyperparameters were evaluated, and the set that provided the best average performance across the 5 folds was selected as the optimal set. Subsequently, we executed models in parallel across each fold and independently calculating the test MSE or test accuracy. The performance metric was then collected and averaged from this parallelized execution to facilitate the Bayesian Optimization process using Tree Parzen Estimator (TPE) for parameter suggestions. The best hyperparameters were selected based on the loss criteria (i.e. MSE or accuracy) of the validation set. This iterative process continued until the predefined stopping criterion was reached. For the TPE method, we relied on the stochasticity inherent in draws from the models, ensuring diverse candidate suggestions from one iteration to the next while incorporating new recommendations from BO [54]. To obtain a balance between time consumption and precision of the performance metric results, we set the BO stopping criterion to 1e-5. The experiments were conducted using 5 NVIDIA Tesla V100 GPUs. Each GPU is equipped with 32 GB of HBM2 memory. The initial parameter ranges of the hyperparameters of the models are public available online along with our code.

There are two important hyperparamters in LassoNet: the $l_1$-penalty coefficient $\lambda$ and the hierarchy coefficient *M*, which control the complexity of the fitted model and the relative strength of the linear and nonlinear components, respectively. First, we performed some initial test runs to determine a suitable range of *M* and $\lambda$. For the $\lambda$, we made sure that the initial dense model with $\lambda =0$ trained well before starting the regularization path. Then the stepsize over $\lambda$ was implemented following the same strategy as the original paper.

#### Evaluation

For each tuned configuration, ensemble predictions were generated by conducting 10 experiments with different random seeds, and the average results are reported on the test set. For the multi-trait classification task, evaluation metrics include average classification accuracy with standard deviation (stddev), Bries scores and the area under the curve (AUC) with standard deviation. For the regression task, the metrics reported are the test mean squared error (MSE) with standard deviation and the Pearson correlation coefficient *r*, averaged across traits for each dataset.

## Material

### Mice data

The first data of our study is the mice data which is part of the BGLR package in R [55], but originally comes from the Wellcome Trust (http://gscan.well.ox.ac.uk) and has been used for whole-genome regression in several other studies [56, 57]. It consists of genotypes and phenotypes of 1,814 mice. Each mouse was genotyped at 10,346 single nucleotide polymorphisms (SNPs) that were coded as 0, 1 and 2. Here we use two continuous traits, body length (BL) and body mass index (BMI). The entire dataset was divided into a training set (70%), a validation set (10%) and an independent test set (20%).

### Pig data

The largest tabular data set in our study is the pig data [58] which contains 3534 individuals with high-density genotypes and continuous phenotypes of five anonymized traits. After cleaning some missing data, we finally obtain 2314 samples and each sample contains 52, 843 SNPs. The data was anonymised by randomising the map order and cording of the SNP genotypes were 0, 1, and 2. The dataset was partitioned into three subsets for training, validation and independent testing using the same approach as for the mice data.

### Wheat data

The wheat data set originates from CIMMYT’s Global Wheat Program and is also a part of the BGLR package [55]. It comprises 599 wheat lines from the CIMMYT Global Wheat Program evaluated in four international environments representing four basic agroclimatic regions (mega-environments). The wheat lines were genotyped using 1,447 Diversity Array Technology (DArT) markers. As a quality control, all the markers with a minor allele frequency below 0.05 were eliminated, and any missing genotypes were imputed using samples from the marginal distribution of marker genotypes. Following these procedures, the dataset was reduced to 1,279 DArT markers which are coded as 0 and 1. We used the data from the different environments as multiple traits, resulting in a total of four traits. The whole datasets was divided into three datasets for training, validation and testing following the procedure of the pig and mice data.

### 14-Cancer microarray data

The cancer data originates from a study by [59] and has been used in for example [48]. The data uses oligonucleotide microarrays containing 16,063 oligonucleotide probe sets for the gene expression. It contains 16,063 gene expression feature values and 198 tumor samples, which were divided into 144 training samples and 54 test samples. Of the training samples, 100 were used for training and 44 allocated for validation. Each feature represents the expression level of a specific gene across various samples. The binary traits constitutes 14 common human cancers, including Breast (BR), Prostate (PR), Lung (LU), Leukemia (LE), Renal (RE), Pancreas (PA), Ovarym (OV), Mesothelioma (ME) and CNS cancers. For further details see [59].

### Loblolly pine data

The lobolly pine population is derived from 32 parent trees representing a wide range of accessions from the Atlantic coastal plain, Florida, and the lower Gulf of the United States. Parents were crossed in a circular mating design with additional off-diagnal crosses, resulting in 70 full-sib families with an average of 13.5 individuals per family [60, 61]. It was originally composed of 951 individuals from 61 families that was genotyped using an Illumina Infinium assay [62]. A subset of 4,853 SNPs (encoded as 0, 1, 2) were polymorphic and used in our study. By discretizing the values of the deregressed breeding values > 0 to 1 and deregressed breeding values < 0 to 0, we recreated two binary traits: presence or absence of rust (Rustbin) and presence or absence of roots (Rootnumbin). Then the traits were recoded to four multi-classes ([0,0],[0,1],[1,0] and [1,1]). After cleaning some missing data, we finally got 806 samples and each sample contains 4,853 SNPs. The dataset was divided into three subsets-training, validation and testing - using the same percentage as for the mice, pig and wheat data.

## Data Availability

The code for the tabular deep learning models and the datasets are available at https://github.com/angelYHF/Tabular-deep-learning-for-GWP. The original data sets are available at: Mice data: https://cran.r-project.org/web/packages/BGLR/index.html Pig data: https://academic.oup.com/g3journal/article/2/4/429/6026060/4294_ FileS1.zip Wheat data: https://cran.r-project.org/web/packages/BGLR/index.html 14-cancer microarray data: https://www.kaggle.com/datasets/tranthinhuy/14cancermicroarraydata Loblolly pine data: https://academic.oup.com/genetics/article/190/4/1503/6064084/ Loblolly_Pine_Resende_.zip.
